# Development of Locomotor-Related Movements in Early Infancy

**DOI:** 10.3389/fncel.2020.623759

**Published:** 2021-01-21

**Authors:** Arthur H. Dewolf, Francesca Sylos Labini, Yury Ivanenko, Francesco Lacquaniti

**Affiliations:** ^1^Department of Systems Medicine, Center of Space Biomedicine, Faculty of Medicine and Surgery, University of Rome Tor Vergata, Rome, Italy; ^2^Laboratory of Neuromotor Physiology, IRCCS Santa Lucia Foundation, Rome, Italy

**Keywords:** early development of locomotion, locomotor precursors, complexity and flexibility of CPGs, sensory modulation of movement, early responsiveness

## Abstract

This mini-review focuses on the emergence of locomotor-related movements in early infancy. In particular, we consider multiples precursor behaviors of locomotion as a manifestation of the development of the neuronal networks and their link in the establishment of precocious locomotor skills. Despite the large variability of motor behavior observed in human babies, as in animals, afferent information is already processed to shape the behavior to specific situations and environments. Specifically, we argue that the closed-loop interaction between the neural output and the physical dynamics of the mechanical system should be considered to explore the complexity and flexibility of pattern generation in human and animal neonates.

## Introduction

Locomotor function bridges the entire life span but its development during fetal age and the first post-natal years of life is crucial for the acquisition of mature behavior. Where does locomotor behavior start? This question, suggesting a developmental continuity, is a central and long-standing issue (Adolph et al., 2011). Continuity supports the idea that new skills grow from the seeds of prior precursors. A line of evidence is the fact that primitive muscular control patterns observed in neonates are highly preserved and recombined during development (Dominici et al., 2011; Sylos-Labini et al., 2020). However, how the different locomotor precursors develop and to what extent they depend on interactions among many subsystems, from individuals’ intrinsic characteristics and their environment, remains, undoubtedly, incompletely understood.

**Figure 1:**
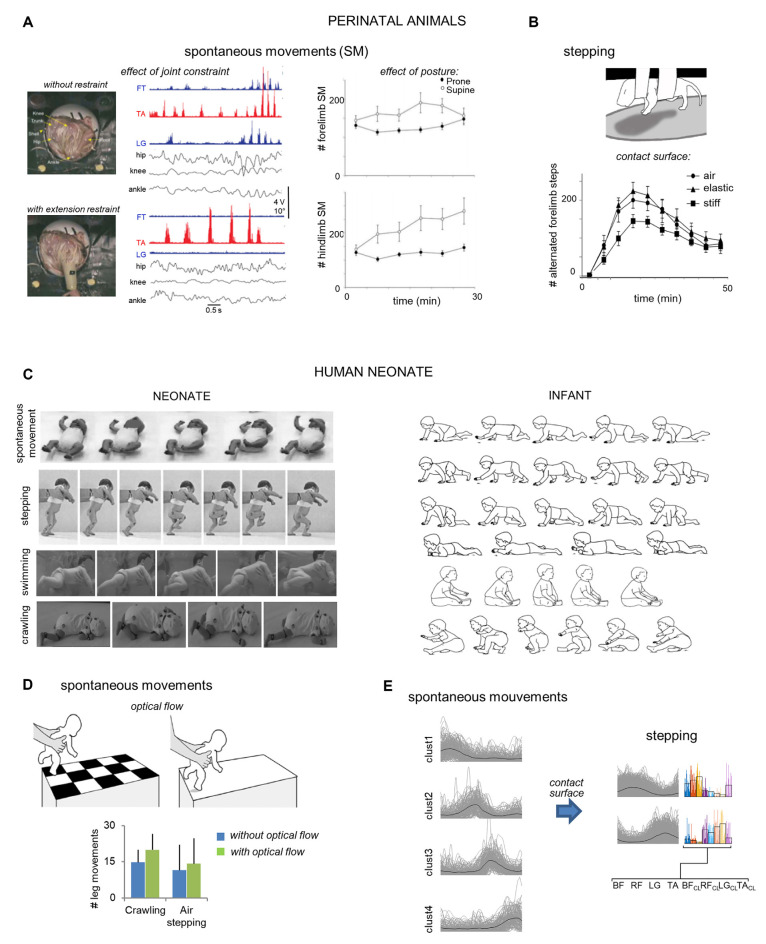
Early responsiveness to sensory or environmental changes in animal **(A,B)** and human **(C,D)** neonates. **(A)** Left: effect of ankle extension restraint, imposed by a lightweight splint, on EMG activity in chicken embryos. During extension restraint, the ankle flexor (TA) was rhythmically active but bursts were longer in duration and larger in amplitude. Also, knee extensor (FT) and ankle extensor (LG) activity dropped out (adapted from Bradley et al., 2014). Right: a greater number of SMs of newborn rat pups in supine than in prone position (from Mendez-Gallardo et al., 2016). **(B)** A smaller number of alternated forelimbs steps induced by drug treatment (quipazine) on a stiff substrate as compared to an elastic or no substrate (adapted from Brumley et al., 2012). **(C)** Left: video frames with examples of different movements of neonates [spontaneous movements (adapted from Hadders-Algra, 2004), stepping (adapted from Domellöf et al., 2007), swimming (adapted from 3s Doodles., 2016), crawling (adapted from Forma et al., 2019)]. Right: illustration of example crawling styles in infants of 10.4 ± 1.5 months old (adapted from Patrick et al., 2012). All sequences start with initiation of stance in the left leg (from top to bottom: standard crawling, hands-and-feet, crawling, step-crawl mix, creeping, and scooting step-scoot mix). **(D)** Increase of air-stepping (adapted from Barbu-Roth et al., 2014) and crawling (adapted from Forma et al., 2018) leg movements in neonates exposed to an optic flow with a pattern moving away from the neonate. **(E)** Effect of the absence (SM) and presence (stepping) of surface contact on muscle coordination (determined using cluster analysis of basic muscle modules). SMs are associated with four basic temporal activation patterns without stable systematic muscle synergies. In contrast, stepping is associated with fewer temporal basic patterns structured in stable synergies. Single cycles of all subjects are plotted in gray, and corresponding weights in color (average patterns in black; adapted from Sylos-Labini et al., 2020).

Humans start to walk significantly later than most animals (Garwicz et al., 2009), and infants discover an array of idiosyncratic solutions for mobility (Patrick et al., 2012; Figure 1C) before having sufficient axial and limb muscles strength and balance control to walk (e.g., McGraw, 1945; Thelen and Ulrich, 1991; Bril and Breniere, 1992; Guillaud et al., 2020). While strategies such as crawling or cruising are still widely depicted in modern “milestone” and assessment charts (Piper and Darrah, 1994; Adolph and Robinson, 2013; Adolph et al., 2018), infants often deviate from common trajectories and develop individual differences in development (Adolph et al., 2011; Atun-Einy et al., 2012). Conversely, in early infancy, precursory forms of spontaneous movements (Figure 1C) appear as more obvious obligatory stages in the development of locomotion.

In neonates, it is commonly thought that human locomotor development stems from single precursor behavior, consisting of alternating flexor–extensor movements (Thelen and Fisher, 1982; Yang et al., 1998). However, Sylos-Labini et al. (2020) recently challenged this view by showing that the neuromuscular control of stepping and spontaneous air movements (also called kicking), two movements displayed in neonates, differ substantially. In particular, these authors suggested that the two behaviors may depend on a dynamic reconfiguration of the underlying neural circuits (Marder et al., 2014) as a function of sensory and mechanical feedback (Duysens et al., 2000; Pearson, 2004).

The major consideration of this mini-review is the existence of multiples precursor behaviors of locomotion as a manifestation of distinct locomotor antecedents, challenging the widespread idea that neonate behaviors all emerge from the same neuromuscular substrate (Thelen et al., 1981). Besides, we also consider a recent interdisciplinary approach to investigating the early plasticity of those motor behaviors. In a final section, we discuss how maturation and early motor experience may shape the control of motor systems, stemming in part from a functional reorganization of intraspinal locomotor circuits (Barbeau and Rossignol, 1987), in different environmental contexts and how it could potentially lead to improved strategies for promoting locomotor function recovery.

## The Manifestation of Multiple Locomotor-Related Movements in Neonates

Neonates also express a range of early locomotor-related movements such as kicking, stepping, crawling, or swimming (Figure 1C). These behaviors present striking similarity with their mature forms observed during adulthood (Andre-Thomas and Autgaerden, 1966; MacLellan et al., 2012), which have fuelled speculation about the idea that precursory forms are actually prerequisites of adult locomotion.

The most studied locomotor-related movement and its developmental continuity is stepping. Neonate babies step on the ground if supported (Thelen and Fisher, 1982; Forssberg, 1985; Yang et al., 1998; Domellöf et al., 2007; Dominici et al., 2011; Adolph and Robinson, 2013; Sylos-Labini et al., 2017, 2020), and stepping generally disappears a few weeks after birth unless trained. The potential relationship between this early behavior and adult walking gait was first suggested by Andre-Thomas and Autgaerden (1966). More recent works have supported the idea that muscle activation patterns of stepping are preserved and recombined through development, suggesting that gaits may be built starting from common conserved elements (Dominici et al., 2011; Dewolf et al., 2020; Sylos-Labini et al., 2020).

Potential other precursors of locomotion have received far less attention than stepping, most likely because of the commonly held view claiming that human locomotor development stems from single precursor behavior, consisting of alternating flexor–extensor movements (Thelen and Fisher, 1982; Domellöf et al., 2007; Barbu-Roth et al., 2014). Following Thelen et al.’s (1981) suggestion, based on spatial and temporal kinematic structure, other authors have speculated that early crawling, swimming, or spontaneous movements are all identical behaviors generated by the same neural mechanisms (Adolph and Robinson, 2013; Barbu-Roth et al., 2014; Forma et al., 2018), failing to depict the potential diversity of precursory forms.

Recently, Sylos-Labini et al. (2020) challenged this influential idea, starting from the premise that the animal neonatal spinal cord can generate a variety of different motor activities (Klein et al., 2010; Hägglund et al., 2013; Machado et al., 2015), and that human neonates can likely do the same. To address this question, they compared the motor patterns of stepping and spontaneous kicking in neonates. In contrast with stepping, spontaneous kicking is produced thousands of times before birth and persists over several months after birth (Thelen and Fisher, 1982; de Vries et al., 1982). Sylos-Labini et al. (2020) found that spontaneous kicking and stepping involved neuromuscular modules with different flexibility and complexity. The prevalence and complexity of spontaneous movements suggest that they have a key role in the functional adaptation of spinal sensorimotor circuits to the biomechanics and in engraving an action-based body representation in the spinal cord (Schouenborg, 2010). Besides, Sylos-Labini et al. (2020) also showed that both behaviors anticipate a subset of features that characterizes later development, supporting the idea that they may represent distinct locomotor precursors, both reflecting preparation for adult mature locomotor movements.

The difference in muscle patterns between the two behaviors might also reflect transient adaptations to the different environmental contexts. Indeed, stepping is triggered by contact with the support surface and limb load whereas sensory inputs are not necessary for triggering spontaneous movements. In the next section, we focus on the early responsiveness of multiple precursors of locomotion to the environment and sensory feedback. Understanding how such factors shape locomotor-related movements may have important clinical implications in infants with developmental neuromuscular disorders.

## Early Modulation of Locomotor Precursors

The rhythmic nature of locomotor precursors involves phasic activation of muscles, resulting from the interplay between the activity of spinal central pattern generators (CPGs), sensory signals originating in the limbs, and supraspinal signals (Grillner, 1981; Büschges et al., 1995). The CPG is a remarkable network of spinal interneurons responsible for producing the fundamental neural commands underlying basic locomotion (Kiehn, 2006; Guertin, 2009; Grillner and El Manira, 2019). Early experiments on animals showed that the CPGs produce basic, phasic locomotor activity, independent of sensory inputs (Brown, 1911, 1914). However, the continuous process of sensory input also plays an important role in modulating spinal networks shortly before and after birth (Brocard et al., 1999, 2003; Brumley et al., 2017), permitting early adaptation to the environmental context. For example, Bradley et al. (2014) analyzed the interactions between the environment and movement experience before hatching in chicks, to determine whether proprioception circuits can modulate leg muscle activity during spontaneous limb movements. To this end, leg muscle activity and kinematics were recorded in embryos without and with an ankle extension restraint (Figure 1A). The extension restraint produced excitation of the ankle flexor and inhibition of the ankle extensor. Therefore, the authors proposed that proprioceptive stimulation from muscle and Golgi tendon organ receptors already contributes to regulating muscle activity during precocious locomotor-related movements. Similar results were already observed in spinal cats, where activation of group Ia and group Ib afferents from ankle extensors prolongs extensors bursts and inhibits flexor activity (Pearson et al., 1992; Guertin et al., 1995).

During the early postnatal period, the locomotor-related movements also exhibit remarkable plasticity (Altman and Sudarshan, 1975). In rats, a series of studies demonstrated how neonates adapt their locomotor behavior to environmental context (Figures 1A,B). For example, Mendez-Gallardo et al. (2016) explored the role of posture in the expression of spontaneous limb movements and showed that rat pups expressed more spontaneous activity while supine than prone (Figure 1A). Contrarily, more stepping was observed in the prone position, suggesting that posture affects the expression of different behaviors during early development. Cutaneous and proprioceptive feedback also modulates the stepping behavior in neonate rats (Figure 1B). Indeed, pups made fewer steps when their feet were in contact with a stiff substrate vs. an elastic substrate (Brumley et al., 2012). Even olfactory sensory inputs can impact locomotion development since locomotor-like rhythmic movements in neonate rats can be also elicited using an olfactory stimulus (Fady et al., 1998). Together, these studies are suggestive of an important role for sensory afferent feedback in the early development of the locomotor system, permitting locomotor adaptations to environmental perturbations.

Based on the striking similarities in the early development of locomotion across mammalian species (Garwicz et al., 2009; Dominici et al., 2011; Grillner, 2011; Yang et al., 2015), similar early modulation of locomotor precursors may be expected in the early postnatal period. While several studies have reported that infant stepping is surprisingly adaptable to a range of different factors (Thelen et al., 1982; Thelen and Ulrich, 1991; Jensen et al., 1994; Pang et al., 2003; Yang et al., 2005), the responsiveness of neonates locomotor precursors has been hardly considered. Recently, Hym et al. (2020) have demonstrated that the circuitry underlying locomotor-related movements in neonates is already adaptable to olfactory inputs, influencing locomotor control centers. Two other studies have shown that neonates are already responsive to visual optic flow (Barbu-Roth et al., 2014; Forma et al., 2018). In particular, these authors showed an increase in spontaneous leg movements during air stepping and crawling when babies are exposed to optic flows (Figure 1D), highlighting surprisingly precocious responsiveness to visual stimulation.

More recently, Sylos-Labini et al. (2020) suggested that the different set of fundamental patterns of muscle activation they observed between spontaneous movements and stepping may depend on the absence or presence of sensory feedback from surface contact (Duysens et al., 2000; Pearson, 2004; Musselman and Yang, 2007). In particular, they observed that neonate spontaneous movement showed activation patterns with a similar dimensionality and waveform as those of mature locomotion, which lacked a stable association with systematic muscle synergies across movements. In contrast, stepping was associated with fewer temporal patterns all structured in stable synergies whose fractionation could account for the synergies of more mature walking (Figure 1E), consistent with the CPG “drive pulse” rhythmic elements in the spinal circuitry of vertebrates (Rauscent et al., 2006; Giszter et al., 2010). Therefore, the authors proposed that the sensory signals generated by the contact with the support during stepping modify the expression of neuromuscular modules, depending on a dynamic reconfiguration of the underlying neural circuits.

To date, numerous experimental paradigms examine the development and early plasticity of locomotion *in vitro*, called fictive locomotion (e.g., Matsushima and Grillner, 1992; Lafreniere-Roula and McCrea, 2005). The termed “fictive locomotion” means that, although the pattern of activity recorded is locomotor-like, it is not real locomotion due to the absence of movement and peripheral sensory feedback. While such an approach helped to identify cellular properties and genetic regulation of CPGs, fictive locomotion cannot reveal the interactions among factors that may influence ongoing behavior, such as the movement-adaptability to sensory feedback highlighted in this section (Figure 1). More behaviorally relevant future studies on the developmental changes and early plasticity of multiple locomotion precursors may open new avenues for developmental improvements and pediatric neurorehabilitation strategy.

Even if the afferent information is used to a limited extent to adapt motor behavior to specific environmental contexts in neonates (Hadders-Algra, 2018), early postnatal behavioral modulation could already be used as a new form of quantitative neuromotor assessment. Indeed, investigating the multiple locomotor precursors during early infancy can help early diagnosis of infants with developmental disorders (Hadders-Algra, 2004). Also, understanding what factors influence and modulate the expression of neonate locomotor movements may have important clinical implications because rehabilitation is likely to yield large benefits when initiated as early as possible. In the next section, the effect of age and experience on the modulation of locomotor-related movements in infants is discussed.

## The Pivotal Role of Experience and Maturation on The Developmental Process

**Figure 2:**
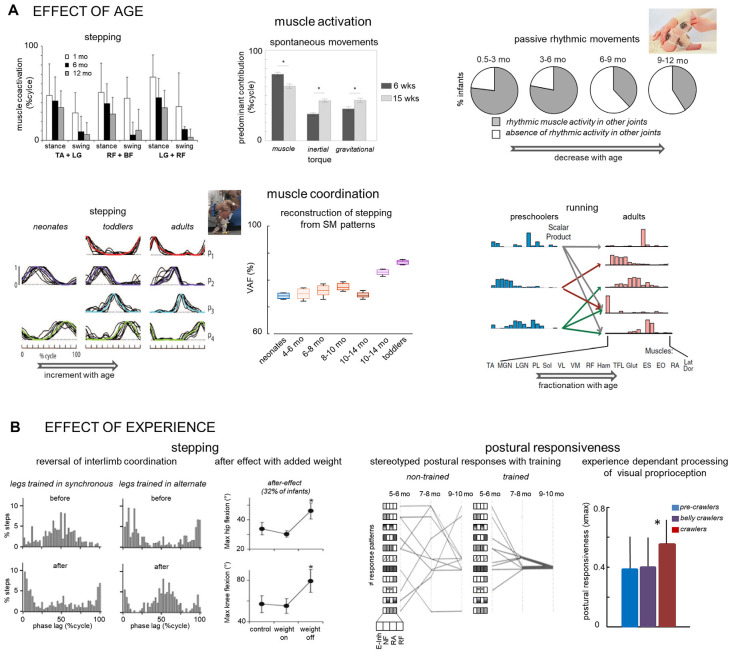
Early development of locomotor-related movements: effect of age **(A)** and experience **(B)**. **(A)** *Upper panels*—age-related changes in *muscle activation* (from left to right): reduction of agonist-antagonist co-activation (adapted from Teulier et al., 2012); change in the differential cofntribution of distinct components (muscle, inertial, or gravitational) of the net knee torque during SMs (adapted from Sargent et al., 2015); reduction of muscle responses in other joints (not being rotated) during passive rhythmic movements (adapted from Solopova et al., 2019). **(A)** *Lower panels*—age-related changes in *muscle coordination* (from left to right): increase of the number of basic muscles activation patterns (adapted from Dominici et al., 2011); reconstruction of stepping muscle activity from basic activation patterns of neonate SMs increases with age (adapted from Sylos-Labini et al., 2020); fractionation of preschoolers muscle synergies of running to become multiple synergies in adults (with no running experience; adapted from Cheung et al., 2020). **(B)** Experience-dependant stepping (left two panels) and postural (right two panels) responses in infants. *Left panel*—the reversal of interlimb coordination in two groups of infants initially displaying distinct leg coordination (stepping and hopping) after 4 weeks of reversed leg coordination training (adapted from Musselman and Yang, 2008). *Second panel*—occurrence of aftereffects (changes in the hip and knee flexion during the swing) following removal of an additional weight on the ankle (adapted from Lam et al., 2003). *Third panel*—development of stereotyped response patterns (block diagrams on the left indicate different initial response patterns) during slow forward surface translations in trained infants (adapted from Hadders-Algra et al., 1997). *Right panel*—postural responsiveness to whole-room forward movement is greater in crawlers who experienced optic flow, in comparison to pre-crawlers and belly crawlers who did not experience it. Responsiveness was evaluated as the max cross-correlation (xmax) between the wall movement and the infant’s postural sway (adapted from Anderson et al., 2019). The * indicate significant differences.

The first years of life represent an important phase of maturation of the central nervous system, processing of sensory information, and posture control. Also, many constraints evolve during the first year, such as the muscle-to-fat ratio in lower-limbs (Thelen et al., 1984) or the relative size and weight of the head (Haywood and Getchell, 2009). Figure 2A considers various findings of maturation of the locomotor-related output in infants.

In particular, studies showed considerable changes in muscle activity with age. For example, agonist-antagonist muscle co-activation during stepping decreases as age increased from 1 to 12 months (Teulier et al., 2012; Figure 2A), suggesting better control of limb movements with maturation. Also, as infants grow, they used a more complex pattern of torque component contribution during spontaneous movements (Sargent et al., 2015). Between 6 and 15-weeks of age, older infants decrease the influence of knee muscle torque and better exploit passive dynamics in the coordination of hip and knee motions (Figure 2A). Muscle tone, which is the foundation upon which other locomotor movements are built, also changes with age. Indirectly evaluated from muscle reactions to slow passive cyclic stretching, Solopova et al. (2019) observed that the occurrence of muscle reaction to stretching and shortening significantly decreased throughout the first year of life (Figure 2A). Taken together, these results may reflect the functional reorganization of the motor circuitry during early development, with an important role in optimizing the efficiency of movement (lower co-activation, smaller muscle torque, reduction of muscle tone).

Such spatiotemporal reorganization of the locomotor output has already been investigated during infancy, highlighting how the rudimentary locomotor-related movements of neonates evolve into mature sophisticated ones (Lacquaniti et al., 2012). In adults, the muscle activity patterns of walking can be decomposed into a set of four basic temporals (Ivanenko et al., 2004; Dominici et al., 2011) whereas in neonates two basic patterns were sufficient to accurately reproduce the muscle activity profiles of stepping (Figure 2B). Similarly, during development, the basic temporal patterns of running in pre-schoolers are fractionated into units with fewer muscles in adults (Cheung et al., 2020; Figure 2B). The discharge characteristics of neonates’ motoneurons (Del Vecchio et al., 2020) also suggest a simpler and less flexible control with a significantly higher extent of synchronous activation of motor units than in adults, presumably to compensate for slower and weaker muscles of neonates.

The elements observed in infancy are not discarded but instead become adapted, in parallel with changes in locomotion biomechanics (Dominici et al., 2011; Cappellini et al., 2020; Cheung et al., 2020; Dewolf et al., 2020) and with the neural maturation of central pathways. Interestingly, Sylos-Labini et al. (2020) showed that the spatiotemporal organization of locomotor output observed during spontaneous movements also anticipates some features of walking development. To demonstrate this, the extent to which the muscle activities of stepping could be reconstructed starting from the basic temporal patterns of neonates’ spontaneous movements was quantified (Figure 2B). The quality of the reconstruction improved with age, supporting a developmental continuum of multiple precursors antecedent to locomotion. Since features of stepping neonates are also retained through development (Dominici et al., 2011; Sylos-Labini et al., 2020), it is plausible that various early locomotor behaviors anticipate a subset of features of mature locomotor movements.

The fine-tuning and reshaping of activation patterns of the multiple precursors in the first year of life stems in part from a functional reorganization of interneuronal connectivity, growing integration of supraspinal, intraspinal, and sensory control (Forssberg, 1985; Thelen and Cooke, 1987; Yang et al., 1998). Indeed, many structures of the central nervous system are not mature at birth. For example, early locomotor behaviors change in relation to the maturation of the vestibular system, descending pathways to the spinal cord, or the morphology of the motoneuron (Clarac et al., 1998; Kinney and Volpe, 2018). Besides, the continuous interactions with the environment also play a causal role in driving development, since sensory inputs arising during locomotor movements also guide the organization of neural circuits at spinal and supraspinal levels during development (Khazipov et al., 2004; Schouenborg, 2010). Early specific training may thus change the locomotor-related output in infants and their experience may shape the development and acquisition of skills. The progressive reorganization of activation patterns with age is indeed malleable in infants and turns out to be experience-dependent. Among the variety of locomotor precursors, limb movements can spontaneously be alternate or synchronous (Thelen et al., 1983; Pang et al., 2003; Musselman and Yang, 2007). By practicing for 4 weeks the form of coordination the infant did not exhibit spontaneously, the great majority of them changed their limb coordination to that practiced (Musselman and Yang, 2008; Figure 2B). Balance training can also affect the development of muscle activity patterns in sitting infants (Hadders-Algra et al., 1996, 1997). Among the large repertoire of early patterns are also the patterns later used by the infants, and training facilitated the development of postural adjustments to perturbation, accelerating the experience-based selection of the most complete patterns of synergist activation (Figure 2B). The processing of sensory information that influences ongoing behavior is also experience-dependent. For example, Anderson et al. (2019) showed that the way infants use patterns of optic flow in the peripheral field of view to regulate their postural sway is influenced by their crawling experience (Figure 2B), with higher postural responsiveness in an infant using the crawling style with greater demand on control of balance.

Human infants also appear to be sensitive to transient changes in sensory input and respond to it appropriately (Yang et al., 1998; Pang and Yang, 2000). They can immediately adjust their motor pattern in an organized fashion in response to sustained changes to the mechanical disturbance, such as a modification of leg weight or limb loading (Yang et al., 1998; Lam et al., 2003; Musselman and Yang, 2007). Furthermore, while all infants adapted to an additional load on the leg during stepping, increasing the generation of hip and knee flexor muscle torques, some of them (~32% of infants) exhibited an after-effect (high stepping) in the first step after removal of the weight (Figure 2B). This after-effect was manifested by a greater hip and knee flexion and may indicate that learning had occurred inducing a recalibration of the motor commands. The factors influencing the occurrence of learning are unknown, but their investigation may also be potentially used as a quantitative neuromotor assessment.

Less insight is available on the role of experience in the developmental process in human neonates during the postnatal period. In animals, motor experience during the prenatal and postnatal period has consequent developmental implications (Provine, 1993; Serradj and Jamon, 2016; Pollard et al., 2017). Also in humans, evidence suggests that varied input is conducive for learning. For example, neonates undergoing daily stepping exercise exhibit an earlier onset of an independent walk than untrained infants (Zelazo et al., 1972). Similarly, usual daily training accelerates independent standing (Sigmundsson et al., 2017) and facilitates gross motor development during early infancy (Super, 1976; Hopkins and Westra, 1988). Such impact of early experience suggests that appropriate training could optimize the development of locomotor behavior (Walton et al., 1992; Muir and Chu, 2002; Serradj and Jamon, 2016).

## Conclusion

In this mini-review article, we highlight the need to increase our understanding of the fine-tuning and reshaping of multiple precursors during the development of mature locomotor movements. Since motor coordination in the neonate is already punctuated by developmental plasticity, early responsiveness to environmental context could potentially be used to diagnose developmental disabilities, but also to design and test early therapies. By calling attention to experience-dependent development of the motor system, we hope this will inspire future studies on the control of movement during early development.

## Author Contributions

All authors contributed to the article and approved the submitted version.

## Conflict of Interest

The authors declare that the research was conducted in the absence of any commercial or financial relationships that could be construed as a potential conflict of interest.
